# A Letter from Philadelphia

**Published:** 1861-03

**Authors:** 


### Correspondence.

Messrs. Editors:—I am sorry to say that I can not agree with the tenor of your article on the subject of *Dental Patents*, which appeared in the December Register.

There are some strong points in favor of patents, which have been presented at various times, and others that occur to me, by which I am forced to the conclusion that *the encouragement afforded by the patent laws is powerfully promotive of discoveries and improvements.*

*Gain, profit* or *remuneration*, call it what we may, is, I am satisfied, the chief incentive to discoveries, the attraction and influence which leads, encourages and sustains the discoverer through all his varied researches, experiments and trials, and that supplies renewed vigor for continued effort, until the goal be reached. I therefore assert boldly, as it is my honest conviction, that if the encouragement—the hope of gain,—afforded by a patent be withdrawn, discoveries and improvements will diminish in number and importance; and further, that whatever may be discovered, will be kept locked up in the breast of its owner—a profound secret—the public debarred of its benefits, and it be in danger of dying with its possessor, as has been the fate of many valuable discoveries.

We are told that in the matter of compounding oil colors, some of the old masters possessed a secret by which their pictures retain to this day their pristine beauty and brilliancy of color. No artist of our time will claim such qualities for the colors now in use. The same is asserted of the art of glass staining, that in past ages it existed in greater perfection than now. To come to a more recent period, and to the apparently insignificant, but really important article of *draw plates*, which the reader will understand is used largely in the mechanic arts for drawing out wire. Fragments of plates designed for this purpose are occasionally found, but not often, that are evidently a composition, so soft that they may readily be filed, and the holes in which may be closed up with a hammer, and then with a broach be easily drilled out to the size required. These plates resist the wear of constant use to an astonishing degree, and may be used as long as there is a particle of the metal left, and therefore their economy fully warrants the almost fabulous prices paid for them. The composition of this plate was a secret, as was the compounding of colors and the staining of glass, and now lost to the world, having died with their authors, with many other and more important discoveries, included in the "lost arts," made prior to the existence of patent laws. Now, is it not reasonable to suppose that if all such discoveries had been protected by patents, the world would still be enjoying their benefits?

Patents and copy-rights have often been compared, and to my mind the comparison furnishes a strong argument in favor of the former. Why does an author copy-right his book but that he may enjoy the exclusive sale, and thus profit by his labor? Now, he uses the vernacular, which is not patentable, and he may not impart a new idea, but the venture is his own, the book stands or falls upon its merits or demerits, and the public will buy or not, according to its worth. So it should be with patents; they should find their level, and should receive support only in proportion to their worth. It does no more follow that every thing that is patented is good, than that every thing that is copy-righted is good. Now, no one finds fault with the law of copy-right or with the use made of it—then why should we complain of and oppose patents?

Two things have greatly aided in bringing patents into disrepute in the dental profession: First, the enormous and disproportionate price demanded for privilege to use, which is a sequence of the extravagant claims and expectations of the patentees. Second, the tendency on the part of the profession to buy without due thought and investigation, on the presumption apparently, that every thing that is patented must be good. Now, I hold that the buyer should not find fault with the principle of patents, if he deceive himself or allow others to deceive him in a purchase of this kind, for in such an operation he should take nothing upon trust (and he has a much better opportunity to investigate a patent than a secret process), and if he can not fully satisfy himself that it has advantages, he should not touch it. That there are so many worthless things patented and palmed upon the profession, is mainly owing to the simple fact that purchasers (or dupes) may always be found. The corrective, therefore, is with the profession, who by a proper course, may put a stop to this indiscriminate patenting, which is discreditable, sustaining only those of acknowledged merit.

Methods of operating or manipulating in the surgical department of our profession can not, I am happy to say, as a general thing, be patented; but mechanical contrivances can, and it is in this direction that improvements and discoveries tend, and in which there is a large field and an open door to all who will enter; but remove the strong inducement of profit, and the entries, as well as discoveries, will, I fear, be very limited.

As to the *moral* involved in a learned profession appearing to encourage and sustain professional patents, I can say, that the good of the people, the comfort and happiness of the human family, is the first consideration, and if their interests are served by such a course, as I honestly believe they are, then the stigma, if stigma it be, may well be borne by the profession.

A favorite and much lauded *medical* view of this subject is, that the physician must be self-sacrificing, above, and even hold in contempt the acquirement of "filthy lucre," willing to spend and be spent for the good of suffering humanity. This is the profession, but what is the practice? The young physician, in commencing his practice, finds it necessary to make his charges very moderate, but as his practice increases, and circumstances warrant, his charges increase, and it is his ambition, for the honor as well as the profit, to become the happy recipient of large fees; and still more, as is often the case in large cities, he does not hesitate to compound with the apothecary for a per centage on his prescriptions. It is just so with the dentist; as the extent of his practice will warrant, up goes his prices from one dollar, step by step, to five or even ten dollars as the case may be, for a single filling. Now is not this the general practice, and does it not show that the love of gain has its influence in these, as well as in all other pursuits in life?

In a conversation with a dental acquaintance on the subject of professional charges, the question was asked, "What is the difference ordinarily between a *three* and a *five* dollar filling?" The answer was, "*precisely two dollars.*" And this really is generally the *only* difference, and which fact but adds to the evidence in favor of the theory here advanced.

I do not wish to be understood as opposing the principle of ample remuneration, for I have always contended that that was a matter for the joint consideration of the practitioner and his patients; but I want to show that this beautiful and benevolent idea of self-denial and self-sacrifice, professed by the medical profession, is not carried out in their practice,—that this high and holy devotion to the relief of all "the ills that flesh is heir to," is not wholly uninfluenced by the "almighty dollar," and that, therefore, their professions on the score of motive are fallacious; and that gain, profit or remuneration, is the inspiration, or chief propelling power to human effort and enterprise; and that while we may regret it we dare not deny it.

These are my propositions in brief: 1st. That the hope of gain leads to discoveries, and that this hope is centered in the procurement and protection of a patent. 2d. That patents are preferable to improvements kept secret; first, because the whole public may enjoy its benefits, as it is open for purchase; second, that the buyer may better ascertain its merits before purchasing than he can that of a secret process; and third, that the improvement will never be lost. 3d. That the disposition to patent every trifling thing in the way of improvement may, to a great extent, be corrected by a more discriminate course on the part of the profession, in confining themselves to the support of only those of unquestioned importance, and in discountenancing and otherwise condemning all others.

*Dental Machinery.*—Permit me, on behalf of the profession, to make some acknowledgement of the enterprise and ingenuity of Mr. Henry Snowden, of Baltimore, who, I venture to say, has the most complete and economical manufactory of dental machinery and appliances to be found anywhere.

By the use of steam power, very economically managed, he operates quite a variety of machinery, by which hand labor is saved to an astonishing extent,—the most perfect system of labor-saving in this branch of manufactures I ever saw,—all of which enables him to supply the trade and the profession direct with all the various articles manufactured by him, such as chairs, spittoons, foot-pieces, operating cases, desks, and tables; his much admired and very popular dental lathes and grinding apparatus, of various patterns and prices; also, forceps, pluggers, scalers and excavators; ingot moulds, soldering lamps and an innumerable variety of little things;—indeed, almost every thing in the way of machinery, instruments and tools required by the dentist. Beside, he is constantly devising and bringing out new aids in this line.

Such enterprise as this should not go unrewarded, and it is but justice to thus make public recognition of his valuable services to the material interests of the profession.

*The Register.*—Allow me to congratulate you upon the handsome appearance of your last (February) issue. The Register, I must say, gives satisfactory evidence of energy and enterprise, as well as good taste, on the part of the publisher; and earnestness, unflagging interest and ability on the part of the Editors, all of which are appreciated by the profession, as is shown by the extent and character of its original department and the large subscription list, which I am assured it enjoys.

I like the Register for these reasons, and for its peculiarly agreeable quality of *sociability.* It apparently does not consider it undignified or derogatory to the character of a professional journal, nor think it a sin, to say something calculated to provoke a laugh, or to indulge in familiar intercourse. It is not stiff, starched not stilted; is not tied down to dry disquisitions, dignified drivellings, or frigid formalities, but shows some fire, which gives out warmth, and when I take hold of it, I feel like communing with a friend. May it ever maintain this character, and if peculiar in it, rejoice in the peculiarity. Yours, O. U. C.

PHILADELPHIA, Feb. 15, 1861.

